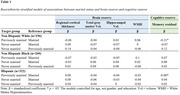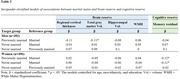# Marital Status and living arrangements, Brain Reserve, and Cognitive Reserve Among Diverse Older Adults

**DOI:** 10.1002/alz.092898

**Published:** 2025-01-09

**Authors:** Ji Hyun Lee, Kiana A. Scambray, Emily P. Morris, Ketlyne Sol, Jordan D Palms, Afsara B Zaheed, Jennifer J. Manly, Adam M. Brickman, Laura B. Zahodne

**Affiliations:** ^1^ Montana State University, Bozeman, MT USA; ^2^ University of Michigan, Ann Arbor, MI USA; ^3^ University of Pittsburgh, Pittsburgh, PA USA; ^4^ The Gertrude H. Sergievsky Center, College of Physicians and Surgeons, Columbia University, New York, NY USA; ^5^ Taub Institute for Research on Alzheimer’s Disease and the Aging Brain, Columbia University, New York, NY USA

## Abstract

**Background:**

Marital status is an important but often overlooked sociodemographic factor that could shape cognitive health in late adulthood. Being married is shown to be linked to lower risk of dementia, but less is understood about underlying mechanisms contributing to this relationship, such as brain reserve (BR) and cognitive reserve (CR). Further, less is known about how living arrangement, independent of marital status, is associated with late‐life cognition. The current study examines (1) associations between marital status, BR, and CR among diverse older adults and (2) whether living arrangement is linked to BR and CR among unmarried adults.

**Method:**

Cross‐sectional data come from the Washington Heights‐Inwood Columbia Aging Project (WHICAP; N = 778, 41% Hispanic, 33% non‐Hispanic Black, 25% non‐Hispanic White; 64% women). Self‐reported marital status was categorized as currently married, previously married (i.e., divorced, separated, widowed), and never married. Magnetic resonance imaging (MRI) markers of BR included regional cortical thickness (in Alzheimer’s disease signature regions), hippocampal volume, gray matter volume, and white matter hyperintensities. CR was defined as residual variance of an episodic memory composite after residualizing MRI markers. Exploratory analyses stratified by race and ethnicity and sex/gender. Age and education was controlled and physical health and income were explored as mediators.

**Result:**

Marital status was associated with CR, but not BR, among White and Hispanic subgroups, and among women, such that those who were previously married had lower CR than their married counterparts. Never married women also had lower CR than married women. Among men only, marital status was associated with BR, but not CR, such that previously married men had lower gray matter volume than married men; however, this finding was not independent of income. Among currently unmarried individuals, living with others was not associated with greater BR nor CR. All findings were independent of age, education, and physical health.

**Conclusion:**

Our findings show differential links between marital status and reserve mechanisms across race and ethnicity and sex/gender, underscoring the importance of examining marital status in older adult’s brain health. Future studies should examine other mechanisms that link marital dissolution and lower CR and nuanced contexts of marital history.